# Intraoperative femurofibular angle combined with tibiofibular angle measurement has fewer correction errors in open-wedge high tibial osteotomy

**DOI:** 10.1186/s13018-024-04619-w

**Published:** 2024-02-19

**Authors:** Chen Zhao, Bing Zhang, Xuejiao Liu, Bo Li, Liang Bao, Cong Liu, Lihong Fan

**Affiliations:** 1 The Second Affiliated Hospital of Xi’an Medical University, Xi’an, 710038 China; 2 The Affiliated Hospital of Northwest University Xi’an No. 3 Hospital, Xi’an, 710016 China; 3 The Second Affiliated Hospital Of Xi’an Jiaotong University, Xi’an, 710004 China

**Keywords:** Alignment line, Femurofibular angle, High tibial osteotomy, Knee alignment, Open-wedge osteotomy, Tibiofibular angle

## Abstract

**Aim:**

This study aimed to verify the accuracy of intraoperative femurofibular angle combined with tibiofibular angle (FFA–TFA) measurement and compare it with traditional alignment line methods in open-wedge high tibial osteotomy (OWHTO).

**Methods:**

A total of 174 knees of 122 patients undergoing OWHTO and using an alignment line or FFA–TFA measurement as an index of optimal correction were included in this retrospective study. The intraoperative alignment line passed through the targeted weight-bearing line (WBL) of the tibial plateau in the alignment line group. The intraoperative FFA–TFA aligned to the preplanned FFA–TFA angle in the FFA–TFA group. WBL, FFA, TFA, and knee joint-line convergence angle of the femur and tibia were assessed as radiological results preoperatively and one year after surgery. The Knee Society Score and the Western Ontario and McMaster Universities were assessed as objective clinical results.

**Results:**

Postoperative WBL in the FFA–TFA group was closer to the target WBL than in the alignment line group (FFA–TFA vs alignment line group: 1.43 ± 1.20% vs 3.82 ± 3.29%; *P* < 0.001). The FFA–TFA group had fewer over-correction and under-correction rates than the alignment line group (28.7% and 12.6% vs 11.5% and 3.40%; *P* < 0.001). No significant differences were observed in the clinical results between the two groups one year after surgery (*P* > 0.05).

**Conclusions:**

The intraoperative measurement of FFA–TFA had fewer complications in terms of under-correction and over-correction compared with the alignment line measurement. No significant differences between the two methods were observed in clinical results one year after surgery.

**Supplementary Information:**

The online version contains supplementary material available at 10.1186/s13018-024-04619-w.

## Introduction

Open-wedge high tibial osteotomy (OWHTO) shifts the lower limb alignment from the diseased medial compartment to the relatively healthy lateral compartment [1, 2], thus postponing knee replacement in patients with medial compartment lesions and varus deformity of the knee [3]. The clinical outcomes after OWHTO depend on the accurate correction of the lower limb alignment according to the preoperative planning [4, 5]. Therefore, proper preoperative planning and accurate intraoperative correction are important for successful OWHTO with optimal long-term benefits [6].

The axial alignment of the lower extremity in OWHTO is influenced not only by the osteotomy angle but also by soft tissue balancing [7, 8]. Intraoperative alignment is widely assessed using an alignment rod or line [9, 10]. However, maintaining consistent lower limb alignment in both weight-bearing and supine positions is challenging due to soft tissue laxity around the knee joint [5]. The change in knee joint-line convergence angle of the femur and tibia (JLCA), which is affected by this soft tissue laxity around the knee after OWHTO, was found to correlate with both the correction amount and correction error [11]. Many previous studies have reported unsatisfactory accuracy using traditional alignment line/rod methods or mechanical medial proximal tibial angle measurement methods in OWHTO [5, 11–13]. The angle between the distal femoral condyle line and the proximal fibula axis line (FFA) has been reported as a preoperative planning tool for OWHTO in a previous study [14]. This study aimed to verify the accuracy of intraoperative femurofibular angle combined tibiofibular angle (FFA–TFA) measurements in OWHTO, comparing it with traditional alignment line methods. Additionally, the study compared clinical results between the FFA–TFA measurement method and the alignment line method. The hypothesis is that corrections made using the FFA–TFA measurement were more accurate than those made using an alignment line.

## Methods

### Study design and patients

**Fig. 1:**
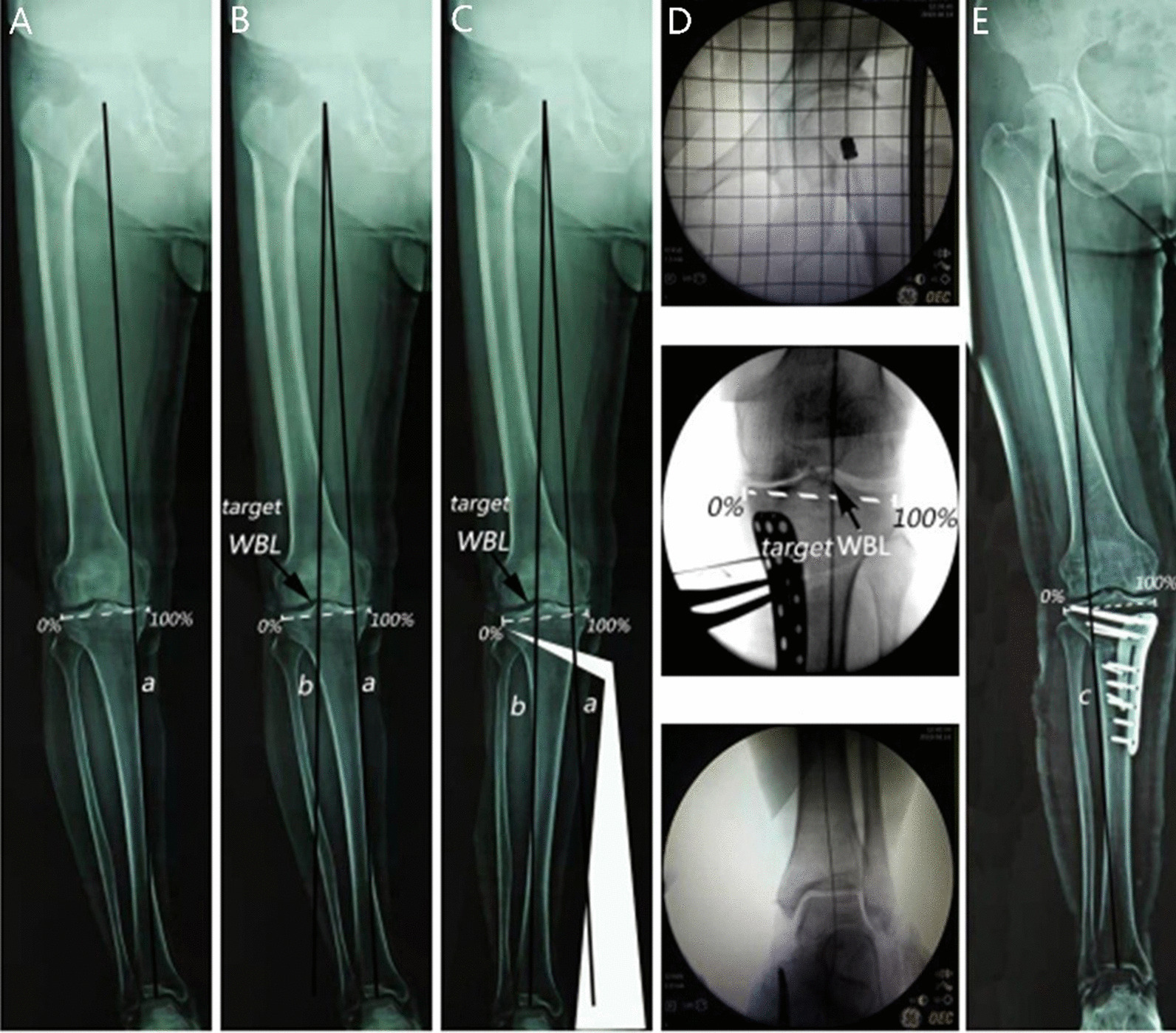
Alignment line group. Preoperative planning (**A**). The preoperative WBL was measured by mechanical axis a. **B** Targeted alignment line b was drawn according to the target WBL. **C** A frame was created and rotated until the ankle joint center was on the targeted mechanical axis b, and the rotation angle of the frame was measured. Surgical correction (**D**). A metal wire was positioned at the center of the hip joint and the ankle joint, confirming that the metal wire passed through the intersecting point of the targeted WBL. Postoperative evaluation (**E**). The postoperative WBL was measured by mechanical axis c one year after surgery

**Fig. 2:**
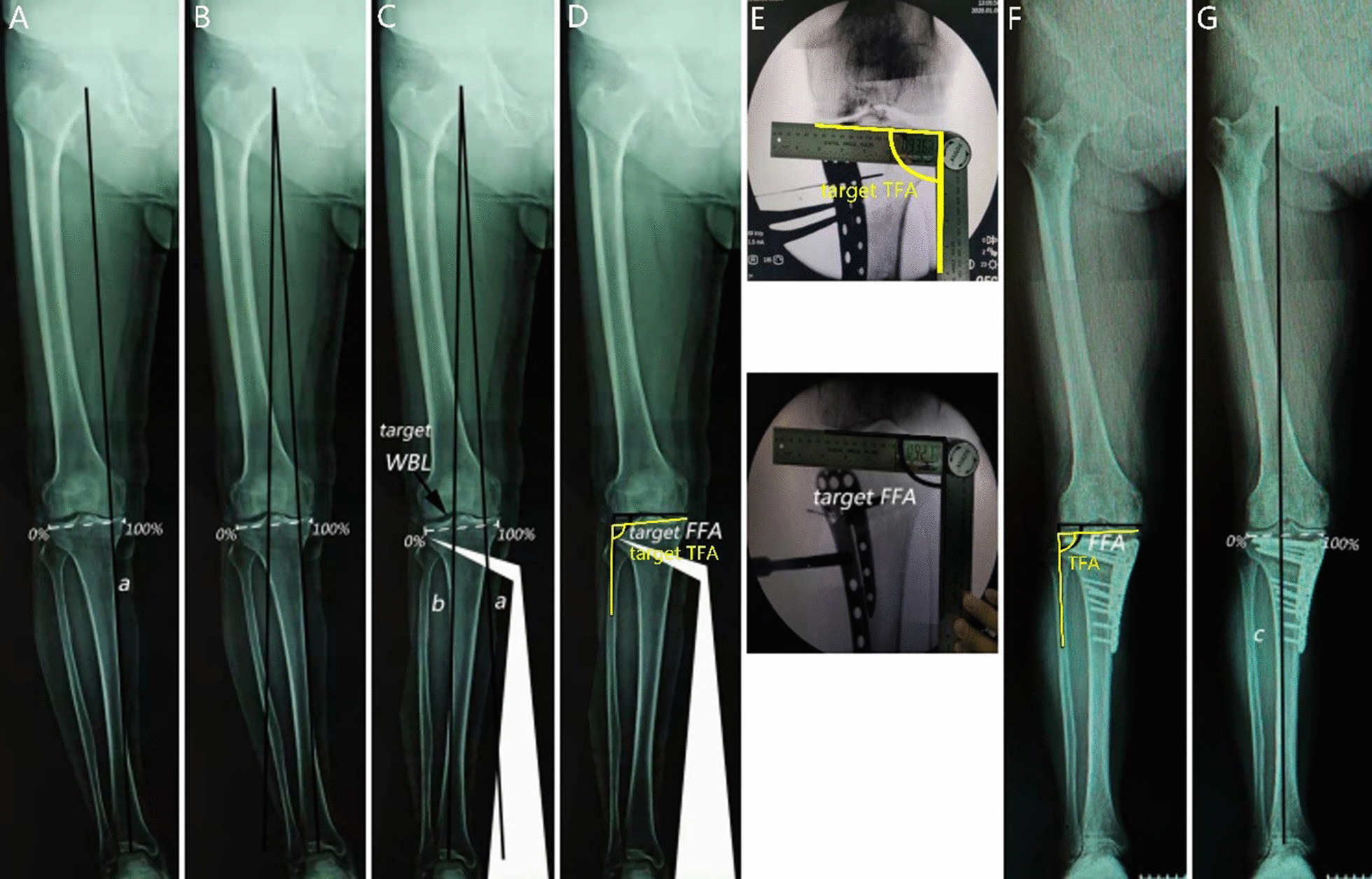
FFA–TFA group. Preoperative planning (**A–C**) was identical to the alignment line group. **D** The angle between the distal femoral condyle line and the tibial plateau line with the proximal fibula axis line was measured as targeted FFA–TFA. Surgical correction (**E**). Confirming the intraoperative FFA and TFA were equal to targeted FFA–TFA. Postoperative evaluation (**F**). The postoperative FFA–TFA was measured one year after surgery. (**G**) Postoperative weight-bearing line (WBL) was measured by mechanical axis c

This study was a retrospective case series. A total of 206 knees of 152 patients undergoing OWHTO for the correction of varus deformity of the knee joint due to osteoarthritis were included in this retrospective study. The inclusion criteria were as follows: (1) age ≤ 70 years, (2) body mass index (BMI) < 30 kg/m^2^, (3) high level of activity, (4) medial knee osteoarthritis ≤ grade III according to the Kellgren–Lawrence (KL) classification, and (5) knee extension loss < 10° and flexion angle > 100°. The exclusion criteria were as follows: (1) symptomatic osteoarthritis of the patellofemoral joint and lateral compartment, (2) rheumatoid arthritis, (3) high-grade ligamentous instabilities, (4) extensive loss or absence of the lateral meniscus, (5) postoperative follow-up time < 12 months, and (6) incomplete follow-up data. From January 2017 to May 2018, an alignment line was used to measure optimal alignment during OWHTO (alignment line group; Fig. 1). From June 2018 to December 2019, the FFA–TFA measurement board (FFA–TFA group; Fig. 2) was used, and optimal alignment was adjusted by matching to preoperatively planned FFA–TFA. Informed consent for the use of medical data was obtained from all patients, and this study was approved by the institutional review board of the Second Affiliated Hospital of Xi'an Jiaotong University (No. X2Y2019-02).

### Preoperative planning

Full-length hip-to-ankle radiographs in a standing position were used to calculate the preoperative weight-bearing line (WBL) ratio at the tibial plateau intersection with the mechanical axis line. Optimal alignment of the lower limb was selected considering meniscus (complex tear and root tear), cartilage (degree and width of the cartilage defect), and KL grade, which usually ranged between 50 and 62.5% [5, 10]. The targeted WBL, degree of correction, and desired opening width were measured using the method previously described by Miniaci [9, 15].

Preoperative OWHTO simulation and measurement of the correction angle and target FFA–TFA were performed using Adobe Photoshop software. First, one line connecting the center of the femoral head and the center of the ankle joint was drawn as the preoperative WBL. Second, a target WBL line connecting the center of the femoral head with the target WBL ratio of the tibial plateau was drawn and then extended to the level of the ankle joint. After that, a frame was drawn to encircle the predicted osteotomy plane from the proximal edge of the tibiofibular joint to the predicted medial osteotomy site, which enclosed the tibia and fibula. The planned osteotomy hinge was taken as the rotation center. The frame was selected, rotated, and moved until the center point of the ankle joint was on the targeted WBL line, and the lateral tibial osteotomy site overlapped from point to point. Finally, the rotation angle was measured as the correction angle, the angle between the distal femoral condyle line and proximal fibula axis line as targeted FFA, and the angle between the articular surface line of the tibial plateau and proximal fibula axis line as targeted TFA (Fig. 2).

### Surgical technique and postoperative rehabilitation

All surgeries were performed by a single orthopedic surgeon with five years of experience in OWHTO. The decision to use OWHTO below the tibial tubercle in the study patients was based on the previously reported advantages, including a greater range of correction, no alteration of patellar height, and more bone stock for rigid fixation [16]. Diagnostic arthroscopy was performed to verify the correct indication before OWHTO. Partial meniscectomy for degenerative tears of the medial meniscus and microfracture analysis for chondral defects of the medial compartment of the knee were performed. Suturing of the meniscus and cruciate ligament reconstruction was not performed in this study.

Two different methods were used to inspect the degree of correction in the two groups during surgery. In the alignment line group, fluoroscopy was used to verify that the electrotome line passed through the center of the femoral head and ankle joint and the electric knife line passed through the targeted WBL ratio of the tibial plateau (Fig. 1). In the FFA–TFA group, fluoroscopy was used to measure the increased FFA and TFA to achieve the targeted FFA and TFA (Fig. 2). The osteotomy was stabilized using a fixed-angle plate with interlocking screws (Π plate, Asia Pacific Medical), and an allogenic bone graft (Jiangsu Shuangyang, China) was inserted into the osteotomy gap. One week after surgery, patients were permitted to begin half-weight-bearing exercises with walker equipment, and full-weight-bearing walking was allowed six weeks after surgery.

### Clinical evaluation

The clinical evaluations were performed for all patients before surgery and one year after surgery. The Knee Society Score (KSS) and the Western Ontario and McMaster Universities (WOMAC) were examined as objective clinical assessments.

### Radiological assessment

**Fig. 3:**
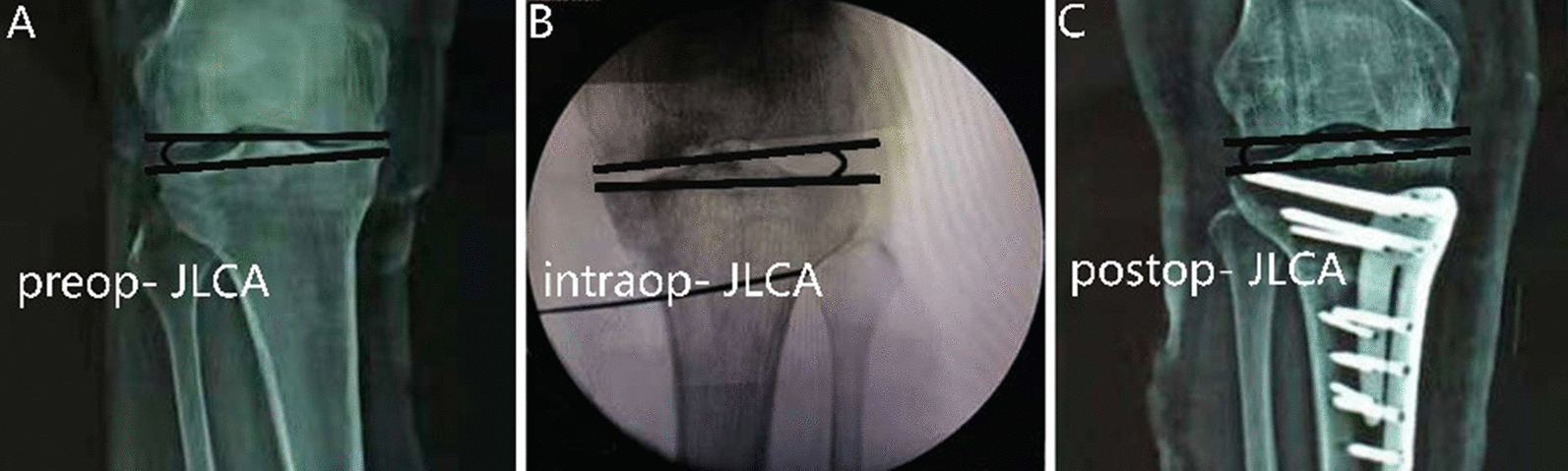
**A** Preoperative JLCA measurement in standing position. **B** Intraoperative JLCA measurement in a supine position. **C** Postoperative JLCA measurement in standing position at one year after surgery

The radiography was carried out under the supervision of a senior orthopedic surgeon. Anteroposterior long-axis radiography in a standing position was performed to assess the preoperative and postoperative radiological parameters (the alignment line group included WBL ratio and JLCA; the FFA–TFA group included WBL ratio, FFA, TFA, and JLCA). Anteroposterior axis radiography of knee joint under supine was performed to assess the intraoperative radiological parameters (the alignment line group included WBL ratio and JLCA; the FFA–TFA group included FFA, TFA, and JLCA) (Figs. 1, 2, 3). The consistency in radiography was achieved using the following criteria: (1) symmetrical shape of femoral and tibial condyles, (2) inter-condylar eminence in the center of inter-condylar fossa, (3) patella in the center of the femoral medial condyle, and (4) overlap of the proximal third of fibular head with lateral tibial condyle. Radiological parameters were independently measured by two examiners (ZC and ZB). The WBL ratio was defined by a line drawn from the center of the femoral head to the center of the superior articular surface of the talus. The width of the tibia measured with a ruler was used as the denominator, and the tibial intersection of the WBL ratio (with a medial tibial edge at 0% and a lateral tibial edge at 100%) was used as the numerator. The correction error was defined as the difference between the targeted and the postoperative WBL ratio, and the obvious over-correction or under-correction was defined as the error of correction ≥ 2.5%.

### Statistical analysis

The intra- and inter-observer accuracies for all measurements were evaluated using the intraclass correlation coefficient (ICC; range: 0–1). Continuous variables were presented as mean and standard deviation (SD). Comparisons among pre-, intra-, and postoperative radiological and clinical results were made using analysis of variance for paired samples. Comparisons among two groups of radiological and clinical outcomes were made using independent-sample *t* test and Chi-square test. The Pearson correlation analysis and logistic regression analysis were used to assess the complications of over-correction and under-correction with JLCA. All data were presented as the means. The *P* values of < 0.05 indicated a statistically significant difference. All statistical analyses were performed using the SPSS Statistic 21.0 software (IBM, CA, USA).

## Results

**Fig. 4:**
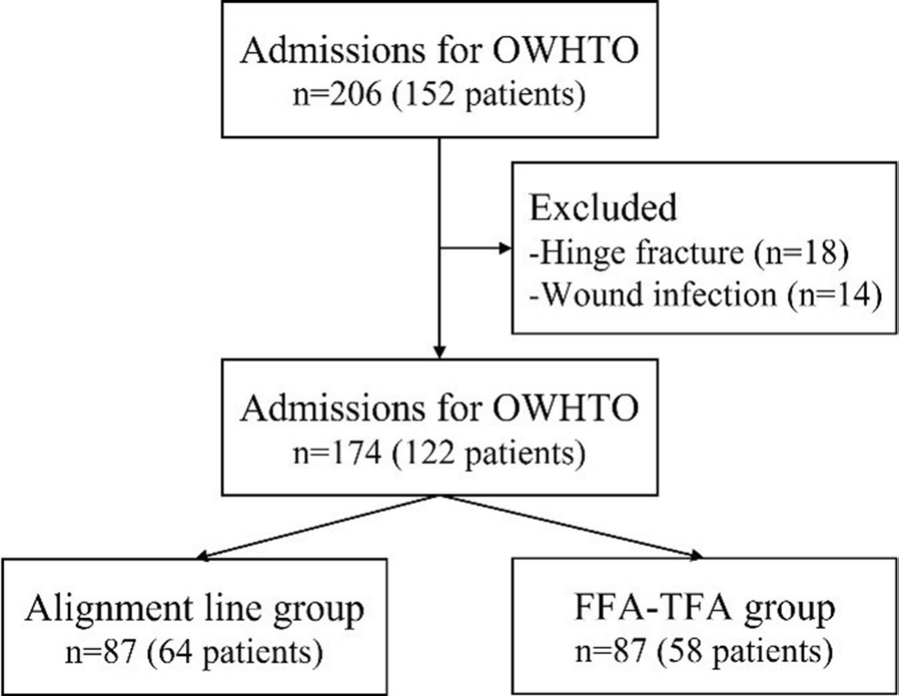
A flowchart showing the algorithm of patient selection

Of 206 knees (152 patients), 18 knees were excluded from the study because of hinge fracture of the proximal tibia, and 14 knees were excluded because their postoperative wound infection delayed rehabilitation. Therefore, 174 knees (122 patients) were available for this study (Fig. 4). The mean follow-up time was 15.57 ± 2.60 months (range, 12–24 months). Further, the alignment line group included 87 knees (64 patients), and the FFA–TFA group included 87 knees (58 patients). No significant differences were observed in terms of age, BMI, and sex ratio between the two groups (Additional file 1: Table S1). The ICC for intraobserver and inter-observer observations ranged from 0.90 to 0.95, indicating almost satisfactory levels of reliability. No statistically significant difference was observed in intra- and inter-observer variabilities before, during, or after the surgery. The outcomes are summarized in Tables 1, 2, 3 and 4.

**Table 1:** Target and postoperative WBL in the alignment line and FFA–TFA groups

	Targeted	Postoperative	Δ_target-postop_	*P*
Alignment line WBL	59.46 ± 3.67	61.22 ± 5.72	3.82 ± 3.29	0.001
FFA–TFA WBL	60.33 ± 3.00	61.11 ± 2.81	1.43 ± 1.20	0.000
*P* value	0.087	0.882	0.000	

All values are presented as the mean ± standard deviation

*SD* standard deviation, *WBL* weight-bearing line

**Table 2:** Preoperative, intraoperative, and postoperative WBL of the alignment line and FFA–TFA groups

	Preoperative	Intraoperative	Postoperative	*P*
*Alignment line group*				
WBL	22.64 ± 16.47	59.50 ± 3.83	61.22 ± 5.72	0.000
JLCA	3.97 ± 1.66	3.66 ± 1.42	3.08 ± 1.26	0.000
*FFA–TFA group*				
FFA	79.34 ± 3.10	89.25 ± 2.90	89.70 ± 2.77	0.000
TFA	83.60 ± 2.77	92.86 ± 2.86	92.57 ± 2.65	0.000
JLCA	4.21 ± 1.43	3.42 ± 1.23	2.99 ± 1.13	0.000

All values are presented as the mean ± standard deviation

*FFA* femurofibular angle, *JLCA* joint-line convergence angle of the femur and tibia, *SD* standard deviation, *TFA* tibiofibular angle, *WBL* weight-bearing line

**Table 3:** Correlation of under-correction–over-correction with JLCA

	Alignment line group					FFA–TFA group				
	*r*	*P*	*β*	OR	*P*	*r*	*P*	*β*	OR	*P*
JLCA_preop-_	− 0.096	0.576	0.836	2.308	0.046	− 0.261	0.389	1.202	3.327	0.294
ΔJLCA_preop-intraop_	− 0.013	0.941	− 1.084	0.338	0.063	0.085	0.781	− 2.198	0.111	0.488
ΔJLCA_postop-intraop_	− 0.590	0.000	1.893	2.308	0.002	− 0.440	0.133	1.778	5.920	0.140

*β*, beta coefficient; BMI, body mass index; FFA, femurofibular angle; JLCA, joint-line convergence angle of the femur and tibia; OR, odds ratio; *r*, Pearson’s correlation coefficient; TFA, tibiofibular angle; WBL, weight-bearing line

**Table 4:** Comparison of clinical results between the alignment line and FFA–TFA groups

	Alignment line group	FFA–TFA group	*P* value
*KSS*			
Before	62.53 ± 4.27	61.76 ± 4.27	0.187
After	90.03 ± 3.94	91.05 ± 2.96	0.057
*P* value	0.000	0.000	
*WOMAC*			
Before	109.16 ± 8.79	108.10 ± 8.41	0.310
After	36.07 ± 6.77	34.54 ± 5.62	0.056
*P* value	0.000	0.000	

All values are presented as the mean ± standard deviation

*KSS* Knee Society Score, *SD* standard deviation, *WOMAC* Western Ontario and McMaster Universities

The postoperative WBL was significantly closer to the target WBL in the FFA–TFA group compared with the alignment line group (*P* < 0.001) (Table 1). Out of the 87 cases in the two groups, there were significantly fewer patients with over-correction and under-correction in the FFA–TFA group than in the alignment line group (*P* < 0.001) (Additional file 1: Table S2).

Intraoperative and postoperative JLCA significantly decreased compared with the preoperative baseline in the two groups (*P* < 0.001) (Table 2). The correction error of alignment was negatively correlated with the ΔJLCA_postop-intraop_ in the alignment line group (*r* = –0.590, *P* < 0.001). A logistic regression analysis indicated that the ΔJLCA_postop-intraop_ (*β* = 1.893, OR = 2.308, *P* < 0.05) were predictors of over-correction and under-correction in the alignment line group. The correction error of alignment was not significant correlation with the ΔJLCA_postop-intraop_ in the FFA–TFA group (Table 3).

At one year after surgery, no significant differences in KSS and WOMAC were observed between the alignment line and FFA–TFA groups (*P* > 0.05) (Table 4).

## Discussion

The present study showed that the intraoperative measurement of FFA–TFA had fewer complications in under-correction and over-correction compared with the alignment line measurement in the OWHTO. FFA–TFA could be used as an index in preoperative planning and intraoperative angle measurement to improve accuracy in OWHTO.

The full-length radiographs in standing positions and Miniaci’s method were usually used for preoperative planning in patients with varus deformity [9]. Despite putting maximum effort into preoperative planning, the preplanned alignment could not be completely achieved in all patients undergoing OWHTO. This was because of the inaccurate preoperative planning of the correction amount and inappropriate intraoperative correction as planned due to a lack of a reliable method for assessing limb alignment during the surgery [17, 18]. Van d et al. reported a systematic review that included nine cohorts using the navigation method in HTO. This review revealed an over-correction rate ranging from 2 to 38% and an under-correction rate from 0 to 23%. Additionally, the review included 14 cohorts employing the conventional method, which demonstrated an over-correction rate from 0 to 16% and an under-correction rate from 0 to 62% in HTO [12]. Our study indicated similar results of an over-correction rate of 28.7% and an under-correction rate of 12.6% in the alignment line group. Obtaining weight-bearing radiographs during the surgery was a challenge. Hence, it was difficult to accurately evaluate the influence of the change in JLCA caused by soft tissue relaxation on mechanical axis lines from surgery to after surgery [19]. In the present study, the JLCA had changed from preoperative to intraoperative to postoperative, and the ΔJLCA_intraop-postop_ was a significant factor correlated with the complication of under-correction and over-correction in the logistic regression analysis. Commonly, after the intraoperative supine position is changed to the postoperative standing position, an excessive increase in JLCA tends to cause under-correction, and an extreme decrease in JLCA tends to cause over-correction after surgery. Lee et al. also proved that the alignment over-correction was related to the significant change in JLCA from before to after HTO [5].

Gil-Melgosa et al. reported that the proximal subluxation of the fibular head from intraoperative supine posture to postoperative standing posture caused the TFA to change in HTO [20]. According to the past literature, the change in JLCA from before to after HTO correlated with coronal correction error, and the JLCA in standing position tends to decrease after surgery [5, 11, 21]. Similar results were found with the results of this study, namely that the postoperative mean angle of JLCA, FFA, and TFA tended to decrease compared with intraoperative measurements in the FFA–TFA group. However, the intraoperative correction using an FFA board tended to be less in patients with over-correction and under-correction compared with that using an alignment line during the surgery, and the correlation between ΔJLCA_postop-intraop_ and the complication of under-correction and over-correction was not significant. Further, the conventional alignment method could result in an inadvertent under-correction or over-correction because the desired mechanical axis is achieved under a fluoroscope, which is needed to confirm the center of the femoral head and ankle joint repeatedly, and the method allowed only for momentary evaluation intraoperatively [22, 23]. The FFA–TFA technology did not require finding the center of the femoral head, and the ankle joint could avoid multiple fluoroscopies to determine the center of the femoral head and ankle joint, which avoided the radiation exposure of doctors and patients and lessen the fluoroscopy times and fluoroscopy error. Taking the abovementioned results together, we can postulate that the intraoperative correction by measuring the targeted FFA and TFA can reduce the impact of JLCA changes and intraoperative fluoroscopy error on the accuracy, thereby reducing the complications in terms of under-correction and over-correction in HTO.

There has been no consensus about an optimal alignment in OWHTO. Early studies suggest that the optimal correction after HTO includes valgus of approximately 8 to 10 degrees in the anatomical axis or 3 to 5 degrees in the MA [24]. Fujisawa et al. reported promising results of cartilage regeneration when the postoperative WBL passed 62%–62.5% of the tibial plateau from the proximal tibial edge, the so-called Fujisawa point [25]. However, one study reported that patients with discoid lateral meniscus were prone to lateral compartment osteoarthritis when HTO was performed using the Fujisawa point [26]. Hohloch et al. reported that the patients with a correction in the areas of 50–55% of the tibial plateau benefited the most compared with the regions of 55–60% and > 60% from the HTO [27]. In our study, the preoperative planning according to individual factors in each patient ranged between 50 and 62.5%, and the short-term clinical symptoms were obviously relieved both in the alignment line and FFA–TFA groups. No significant differences of KSS and WOMAC were observed at one year after surgery between the two groups despite substantial differences in the accuracy of targeted WBL. According to the past literature, it is considered that a personalized preoperative correction plan of alignment leads to a favorable clinical outcome for OWHTO.

## Limitations

This study had several limitations. First, the radiological parameters were measured only in the coronal plane, and the accuracy of intraoperative measurement was limited because of human operation. Second, this case series was a retrospective analysis with only a one-year follow-up period. Third, this study involved two groups that were divided according to the time of the procedure, which may have various biases, including the effect of proficiency level. In future studies, for an accurate comparison, both methods should be measured intraoperatively, and the discrepancy should be examined. Further, using preoperative FFA–TFA for successful medial OWHTO requires careful consideration of several critical criteria. First, the whole knee joint and proximal fibula should be visible via fluoroscopy during surgery. Second, although using Adobe Photoshop software for OWHTO provided accurate simulation, inevitable errors were observed in actual measurement. Therefore, FFA–TFA measurement should be just used as one of the methods for inspecting correction during surgery.

## Conclusions

The intraoperative measurement of FFA–TFA had fewer complications in terms of under-correction and over-correction compared with the alignment line measurement. No significant differences were observed in clinical results one year after surgery between FFA–TFA and alignment line measurement methods.

### Supplementary Information

**Additional file 1. Table S1.** Comparison of demographic data for the alignment line and FFA groups. **Table S2.** Comparison of overcorrection and undercorrection of the alignment line and FFA groups.

## Data Availability

All data generated or analyzed during this study are included in this manuscript and its supplementary information file.

## Abbreviations

FFA–TFA: Femurofibular angle combined tibiofibular angle
JLCA: Joint-line convergence angle of the femur and tibia
KSS: Knee Society Score
OWHTO: Open-wedge high tibial osteotomy
SD: Standard deviation
WBL: Weight-bearing line
WOMAC: Western Ontario and McMaster Universities
