# Supplementary material for: Intraoperative femurofibular angle combined with tibiofibular angle measurement has fewer correction errors in open-wedge high tibial osteotomy
Source: J Orthop Surg Res. 2024 Feb 19;19:148. doi: 10.1186/s13018-024-04619-w (PMC10877754; doi:10.1186/s13018-024-04619-w)
Supplement: Supplementary file 1 — Additional file 1. Table S1. Comparison of demographic data for the alignment line and FFA groups. Table S2. Comparison of overcorrection and undercorrection of the alignment line and FFA groups. [file 13018_2024_4619_MOESM1_ESM.docx]

**Table S1.** Demographic data for the alignment line and FFA-TFA groups

|  | Alignment line group | FFA-TFA group | *P* value |
| --- | --- | --- | --- |
| No. of knees/patients | 87/64 | 87/58 | – |
| Age (year), mean ± SD (range) | 63.33 ± 7.51 (46–70) | 62.91 ± 6.83 (47–70) | 0.697 |
| Male, *n* (%) | 34 (53%) | 28(49%) | 0.273 |
| Body mass index (kg/m^2^), mean ± SD (range) | 25.71 ± 3.18 (19.1–34.0) | 26.07 ± 2.84 (21–33) | 0.448 |
| Kellgren–Lawrence grade, *n* (%) |  |  | 0.153 |
| II | 35 (40%) | 26 (30%) |  |
| III | 52 (60%) | 61 (70%) |  |
| WBL (%), mean ± SD (range) | 22.64 ± 16.47 (–12.65 to 50.24) | 23.35 ± 15.56 (–9.64 to 49.97) | 0.772 |

All values are presented as the mean ± standard deviation; SD, Standard deviation; WBL, weight-bearing line.

**Table S2.** Over-correction and under-correction of the alignment line and FFA-TFA groups

|  | Over-correction  (the error of correction ≥2.5%) | Under-correction  (the error of correction ≥2.5%) | *p* |
| --- | --- | --- | --- |
| Alignment line group  (n=87) | 25(28.7%) | 11 (12.6%) | 0.000 |
| FFA-TFA group  (n=87) | 10 (11.5%) | 3 (3.4%) |  |

All values are presented as the mean ± standard deviation. SD, Standard deviation.
